# Effectiveness and Tolerability of a Patch Containing Onion Extract and Allantoin for Cesarean Section Scars

**DOI:** 10.3389/fphar.2020.569514

**Published:** 2020-09-25

**Authors:** Valeria Conti, Graziamaria Corbi, Teresa Iannaccone, Bianca Corrado, Luigi Giugliano, Serena Lembo, Amelia Filippelli, Maurizio Guida

**Affiliations:** ^1^ Department of Medicine, Surgery and Dentistry “Scuola Medica Salernitana”, University of Salerno, Baronissi, Italy; ^2^ Department of Medicine and Health Sciences, University of Molise, Campobasso, Italy

**Keywords:** natural products, onion extract, allantoin, wound healing, cesarean delivery

## Abstract

**Background:**

The prevention or early treatment of pathological scars is the most appropriate therapeutic approach. Gels and patches containing onion extract and allantoin are safe and effective in patients with scars of various origins and severity. However, no controlled studies have evaluated the effects of the patch formulation in women after Cesarean delivery. This study aimed to investigate the effects of a patch containing Allium cepa and allantoin on Cesarean section (C-section) scars.

**Methods:**

This is an observational study. Women were consecutively recruited at the University Hospital of Salerno and subdivided into two groups considering the number of C-section. Group A included subjects without and group B with a history of C-section. Scars assessment was made using digital photographs and the Patient and Observer Scar Assessment Scale (POSAS). After 4 weeks, the C-section of the women who had applied a patch containing Allium cepa and allantoin and those of women who had not used any products (controls) were re-evaluated as at baseline. The Observers independently performed the scars assessment at baseline and after 4 weeks. Data are expressed as the difference of the POSAS scores after 4 weeks minus the POSAS scores at baseline. The statistical significance was established at a p value <0.05.

**Results:**

Ninety-three subjects completed the study (47 in group A and 46 in group B). Women who had used a patch showed an improvement in total score by observer scale when compared with controls (p = 0.013). By the patient scale, no significant changes from baseline were found in group A and group B. Group B with patch showed changes in scars’ pigmentation (p = 0.015), relief (p = 0.039), and pliability (p = 0.046) in comparison of controls. Digital photographs confirmed such improvements in women who had already undergone previous C-section, while no significant changes from baseline were found in women without a history of C-section.

**Conclusions:**

Intense treatment of just 4 weeks with a patch containing Alium Cepa extract and allantoin was able to improve pigmentation, relief, and pliability of C-section scars in women with a history of C-section.

**Clinical trial registration:**

ClinicalTrials.gov, identifier NCT04046783.

## Introduction

Wound healing is a dynamic process that occurs in response to trauma, burns or surgical procedures, such as Caesarean section (C-section). It is a very complex process encompassing 4 phases: hemostasis, inflammation, proliferation and remodeling. Several molecules, such as growth factors, cytokines, and agents mediating neo-angiogenesis concur all together to repair the wound, restore skin structure and function and re-establish the underlying circulation (Clark, 2001).

Wound healing inevitably leads to skin scarring, which in turn may cause hypertrophic scars and keloids. The latter represent the most complicated scars because they are unlikely to regress over time (Gangemi et al., 2008).

A detailed assessment of the scar (the acronym for symptoms, color, appearance, and restriction) is the essential step for choosing the most appropriate therapeutic strategy (Gold et al., 2014).

Several scales are available to evaluate the appearance and potential symptomatology of the scars both from clinicians and patients’ perspectives. One of them is the Patient and Observer Scar Assessment Scale (POSAS) (Draaijers et al., 2004; Truong et al., 2007; Fearmonti et al., 2011).

Preventing pathological scars and keloids is more appropriate than treating them because the therapeutic options are often invasive and however not resolutive. Surgical excision, often followed by injection of steroids and occlusive action operated by sheeting or bandages, is the most used treatment in the case of pathological scars. Notably, the recurrence rate after surgery is high, ranging from 40 to 100%, and strongly dependent on the original scar localization (earlobes, sternum, back, etc.) and severity (Gauglitz et al., 2011).

There are several non-invasive remedies useful to both prevention and treatment of pathological scars. Some topical treatments contain natural extracts such as green tea, Aloe vera, vitamin E and D, and onion (Allium cepa) extract useful in contrasting some diseases (Forte et al., 2016; Corbi et al., 2016) and impairments in processes, including wound healing (Prager and Gauglitz, 2018). For many years, a gel containing onion extract and allantoin has been used as anti-scar medications (Karagoz et al., 2009).

Antioxidant, anti-inflammatory, antimicrobial and anti-proliferative activities of the onion extract and moisturizing, anti-inflammatory, and immune-modulatory properties of the allantoin favor a dermo-protective and dermo-repairing action (Mehta et al., 2016).

Then, the same ingredients in the gel have been used in a patch formulation combining the effect of these natural products with an occlusive action on the scar (Prager and Gauglitz, 2018). Many studies have tested different commercial gel formulations demonstrating beneficial effects on scars of various origin and severity, such as post burns and post-surgical scars (Sidgwick et al., 2015). Both gel and patch containing herbal natural products have demonstrated efficacy in guiding to correct scarring over at least 6 weeks (Chuangsuwanich and Jongjamfa, 2014; Prager and Gauglitz, 2018).

The patch formulation combines dermo-restructuring properties of the natural agents and compressive action. It now is commonly used as it is easy to apply encountering the patients’ compliance.

Recently, effectiveness and tolerability of such a device have been examined on post-dermatological surgery scars showing positive effects of such a treatment in promoting scar healing (Prager and Gauglitz, 2018), while it has never been evaluated in women who underwent C-section.

Therefore, the present study aimed to evaluate the efficacy and safety of a patch formulation containing Allium cepa extract and allantoin on C-section scars.

## Materials and Methods

### Setting and Study Population

This is an observational study. Healthy Caucasian women who underwent C-section were consecutively recruited at the University Hospital of Salerno. The inclusion Criteria included: subjects who had undergone Cesarean delivery, age >18, voluntary participation to the study, and informed consent release. The exclusion criteria were represented by age of <18; pre-term birth, obesity, smoking habit, gestational diabetes, hypertension, infections, dermatologic diseases, use of both systemic and topic corticosteroids, and no informed consent release. An identification number was assigned to each subject, and stayed hidden to the observers’ performing the POSAS evaluation.

Taking into account the number of C-section, the study population was divided into two groups: group A included subjects without a history of C-section and group B subjects who had already undergone previous C-section.

At baseline, after stitches removal, scars assessment was made using digital photographs and the validate Patient and Observer Scar Assessment Scale (POSAS).

After 4 weeks, taking advantage of the planned outpatient gynecological visit, women from both group A and B were asked whether had applied some natural products on the scars or not who suggested the use, its mode, and time of administration. Hence, the C-section of the women who had applied a patch containing a standardized quantity of Allium cepa and allantoin and those of women who had not used any products (who represented the control group) were re-evaluated as at baseline. Then, *a posteriori* acquisition of data on the patch use by the women was achieved. Because of *a posteriori* acquisition of the data, the researchers did not provide any instruction to the women that used the patch. The women using the patch reported that had received the suggestion to use the device and the instructions by the cooperating pharmacist in the community. The patch features an occlusive active release liner with an adhesive layer separated by a micro-air cushion seal (Prager and Gauglitz, 2018).

**Figure 1:**
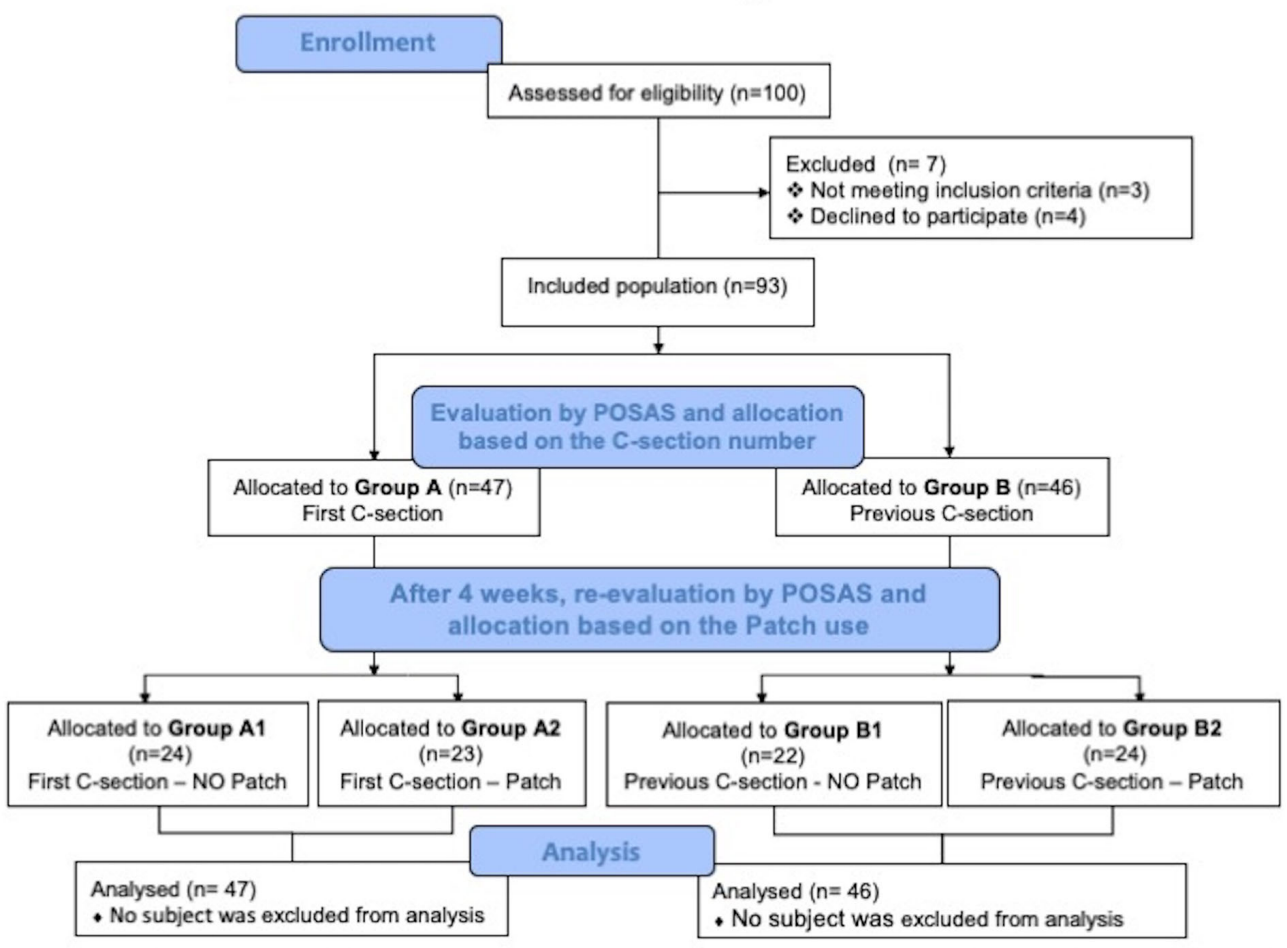
Disposition of women in the study design adapted by the CONSORT algorithm.

Based on the patch use or not, each group was further subdivided in other two groups. At the end, 4 groups were identified: Group A1 including women at the first C-section that did not use the patch, group A2 including women at the first C-section who had applied overnight a patch containing a standardized quantity of Allium cepa extract and allantoin, group B1 including women who have already undergone previous C-section that did not take the patch and who represented the control group, and group B2 including women who have already undergone previous C-section that used the same patch of group A2 (**Figure 1**). All subjects did not use any drug.

Verbal and written information was given to the women and their written informed consent was obtained. The local Ethic Committee “Comitato Etico Campania Sud” approved the protocol of the present study (approved number 27_r.p.s.o). The study was registered under the US NIH ClinicalTrials.gov identifier NCT04046783.

### Assessment Scales and Qualitative Observations

After stitches removal (at baseline), scars assessment was made using digital photographs and the POSAS (version 2.0). The POSAS consists of two parts: a patient and an observer scale, both including six items the sum of which produces a total score. The patient scale contains six questions to evaluate pain, itching, color, pliability, thickness, and relief; the observer scale analyzes vascularity, pigmentation, pliability, thickness, relief, and surface area of the scar. Since to appreciate the difference between pigmentation and vascularity may be particularly difficult for patients, such characteristics were captured in the item “color” (Idriss and Maibach, 2009; Fearmonti et al., 2011). Each item included in POSAS is evaluated by a 10-point score, with “10” indicating the worst imaginable scar or sensation and “1” corresponding to normal skin (normal pigmentation, no itching, etc.). Moreover, the patients also score their “general opinion” on the scar appearance. Two observers were trained in POSAS administration and evaluation. The observers independently performed the scars assessment at baseline and after 4 weeks, unaware of any other information on each subject. Both the observers were blinded on the patch use or not. The match between the POSAS evaluations, linked to the identification number, and the study group, as the analysis of the results, was performed by another independent researcher. The patch was developed in a way to avoid potential indentation on the skin. Moreover, no one of the observers reported any possible mark for the subjects’ identification.

### Statistical Analysis

Data were analyzed using the SPSS (v 23.0) software package (SPSS Inc., Chicago, IL, USA). The sample size was calculated from a similar study including women treated with a patch containing Allium cepa extract and allantoin (Prager and Gauglitz, 2018). We used an estimated standard deviation of 0.5 and the two tailed alpha set at 0.05. An n = 37 per group was able to provide power at 0.9 to detect a significant difference between treatment and control groups. The Shapiro-Wilk Test was used to assess the normal distribution of data. The POSAS observer’ scale scores represented the mean values assigned by the two independent observers. The inter-observer reliability was calculated by computing the intraclass correlation coefficient (ICC) using a two-way mixed model with single measures of consistency. To better define the benefit achieved by the patch administration, a delta calculation (as the difference of the scores after 4 weeks minus the scores at baseline) was used. Differences between multiple groups were evaluated by analysis of variance (ANOVA) with the Bonferroni *post hoc* test and are presented as mean ± SD. The χ2 test was used to compare categorical variables. To explore the correlation between variables, Spearman’s correlation (r) was used. The statistical significance was established at a p < 0.05.

## Results

One hundred subjects who underwent Cesarean delivery were enrolled and 93/100 women (47 in group A and 46 in group B) completed the study. All were Caucasians with age ranging from 21 to 36 (group A) and 26 to 40 (group B). The characteristics of the study population are reported in **Table 1**. The Fitzpatrick skin phototype classification was used (Fitzpatrick, 1975). The women who used the patch referred to have applied it once a day, wearing the device overnight.

**Table 1:** Characteristics of the study population subdivided for the number of C-section and the use or not of the patch.

	Group A1*n = 24*	Group A2*n = 23*	Group B1*n = 22*	Group B2*n = 24*	p
**Age (years)**					
** Mean (SD)**	27.20 (3.19) *	28.44 (4.31)**	33.38 (3.25)	34.33 (5.73)	**0.002**
** 95% CI**	23.23–31.17	26.14–30.73	30.66–36.09	30.70–37.97	
**N. of previous C-section**					
**Means (SD)**	0	0	1.25 (0.46)^	1.25 (0.45)^^	**<0.0001**
** 95% CI**	0	0	0.86–1.64	0.96–1.54	
**Skin phototype, *n* (%)**					**0.585**
** Type I**	0 (0)	0 (0)	0 (0)	0 (0)	
** Type II**	0 (0)	1 (4.35)	1 (4.54)	0 (0)	
** Type III**	24 (100)	22 (95.65)	21 (95.45)	24 (100)	
** Type IV**	0 (0)	0 (0)	0 (0)	0 (0)	
** Type V**	0 (0)	0 (0)	0 (0)	0 (0)	
** Type VI**	0 (0)	0 (0)	0 (0)	0 (0)	
**POSAS patient’ scale total score, *mean (SD)***					
** At baseline**	22.40 (3.29)	21.69 (4.44)	22.75(3.85)	21.25(3.79)	**0.853**
** After 4 weeks**	12.20 (2.49)	12.13 (5.03)	12.12 (4.52)	8.25 (4.47)	**0.122**
**POSAS observer’ scale total score, *mean (SD)***					
** At baseline**	21.80 (4.32)	18.50 (3.93)	25.38 (6.09)	21.75 (8.4)	**0.083**
** After 4 weeks**	14.00 (3.39)	9.63 (4.22)^#^	16.50 (7.07)^##^	8.33 (5.93)	**0.007**

*Group B2 vs. Group A1: p = 0.031; **Group B2 vs. Group A2: p = 0.009; ^ Group B1 vs. Group A: p < 0.0001; ^^Group B2 vs. Group A: p < 0.0001; ^#^Group B1 vs. Group A2: p = 0.030; ^##^Group B2 vs. Group B1: p = 0.011.

In bold are reported the statistically significant p-values.

The analysis was performed taking into account, for each group, whether a patch containing Allium cepa and allantoin was used or not. This led to a subdivision of the study population into four groups: Group A1 (n = 24) including women at the first C-section that did not use the patch and who represented the control group, group A2 (n = 23) including women at the first C-section who had applied overnight a patch containing a standardized quantity of Allium cepa extract and allantoin, group B1 (n = 22) including women who have undergone previous C-section that did not use the patch and who represented the control group, and group B2 (n = 24) including women who had already undergone previous C-section that used the same patch of group A2.

The disposition of women in the study design is shown in **Figure 1**. Discrepancies on the POSAS observer scores interested only the pigmentation and the elasticity scores at baseline, with the ICC of 0.983 (95% CI, 0.969–0.991) and 0.959 (95% CI, 0.925–0.978), respectively.

### Treatment Effectiveness

**Figure 2:**
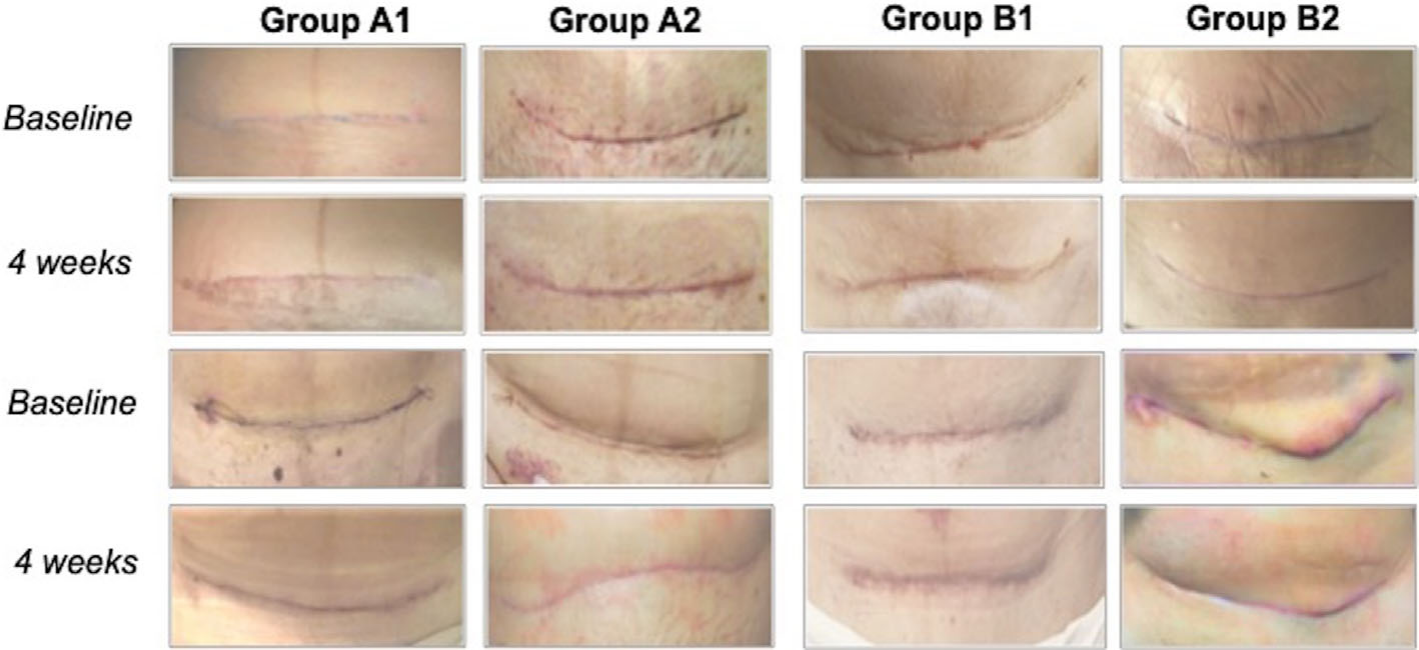
Digital photographs of two subjects for each group at baseline and 4 weeks.

At baseline and after 4 weeks, two blinded observers (independent from each other) assessed the scars of the enrolled subjects by using the POSAS. Digital photographs of two subjects for each group are shown in **Figure 2**. Overall, after 4 weeks, all the recruited subjects showed an improvement in the POSAS scores.

**Figure 3:**
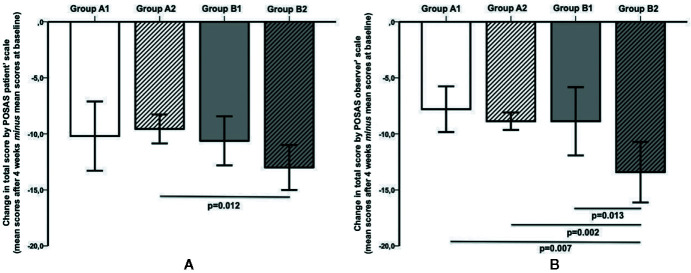
**(A)** The subjects of Group B2 (Previous C-section-Patch) showed a significant improvement (p = 0.012) in the total score according to POSAS Patient scale in comparison with the subjects in group A2 (first C-section-Patch). **(B)** The subjects in Group B2 showed a significant improvement in total score according to POSAS Observer scale when compared with all other groups (vs. group B1 (Previous C-section-NO patch), p = 0.013; vs. group A2, p = 0.002; vs. *g*roup A1 (First C-section- NO Patch), p = 0.007). No other differences were found in Group A.

**Figure 3** shows the patient (**Figure 3A**) and the observer (**Figure 3B**) POSAS total scores. The data are expressed as the difference of the POSAS scores after 4 weeks minus the POSAS scores at baseline. The subjects of the group B2 showed a significant improvement in total score according to the POSAS observer scale when compared with the controls B1 (p = 0.013). Concerning the patient scale, no statistically significant changes between patch and no patch from baseline were found neither in the group A nor in the group B.

**Figure 4:**
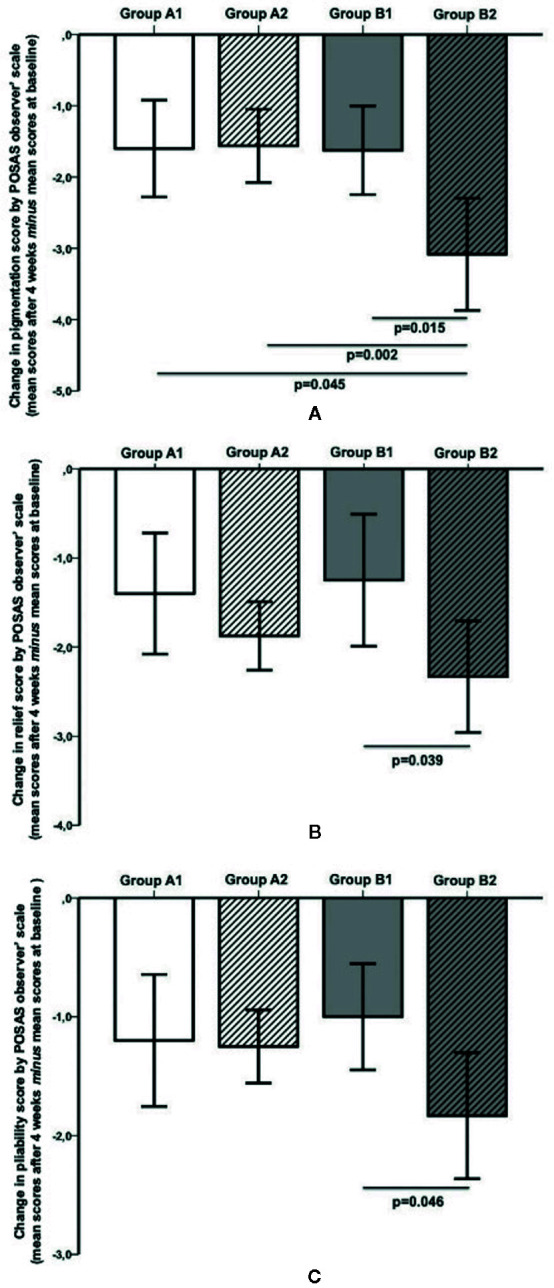
**(A)** The subjects in Group B2 (Previous C-section-Patch) showed a significant improvement in pigmentation according to POSAS Observer scale when compared with all other groups (vs. Group B1 (Previous C-section-NO patch), p = 0.015; vs. *g*roup A2 (First C-section-Patch), p = 0.002; vs. Group A1 (First C-section- NO Patch), p = 0.045). **(B)** The subjects in group B2 showed a significant improvement (p = 0.039) in relief according to POSAS observer scale in comparison with the group B1. No other differences were found in group A and between groups. **(C)** The subjects in group B2 showed a significant improvement (p = 0.046) in pliability according to POSAS Observer scale when compared to the group B1. No other differences were found in group B and between groups.

In group A, no differences in POSAS items were found. Conversely, group B showed statistically significant changes in pigmentation (p = 0.015), relief (p = 0.039), and pliability (p = 0.046) between subjects who had used the patch and controls (**Figures 4A–C**).

The results regarding the other parameters included in POSAS are shown in the supplementary material (**Figures S1–S9**).

### Satisfaction and Safety

The item “general opinion” included in the Patient Scar Assessment Scale was evaluated besides the other POSAS items. The women who had already undergone previous C-section (B2), expressed satisfaction and comfort at the end of the patch use (supplementary material, **Figure S3**), greater than all other groups, whereas without reaching statistical significance. Only one adverse reaction consisting of a light itching that did not require patch discontinuation was reported in one subject in group B2.

## Discussion

The present study showed for the first time that overnight use of a patch containing Allium cepa extract and allantoin for just 4 weeks might ameliorate the overall appearance of C-section scar. Subdividing the study population in groups including women without and with history of C-section, allowed us to compare the effects of the patch in subjects similar from each other but differentiated by C-section number, and to highlight the importance of personalizing the scar treatment and management. Indeed, our results demonstrate that an intense but short-term application of the patch induced significant improvements in pigmentation, relief, and pliability only in women who had already undergone previous C-section. Notably, these three items are all included in the observer-evaluated POSAS.

The wound healing is a process occurring in response to dermal injury. The resulting scar may have various characteristics ranging from fine-line and asymptomatic to hypertrophic scars and keloids. The prevention or early treatment of pathological scars is recognized as the most appropriate approach also to prevent the psychological consequences of the pathological scars that not only cause pain, itching, and other symptoms but also may be so noticeable to lead to emotional distress, affecting the quality of life (Clark, 2001; Gangemi et al., 2008).

Among available remedies, gels and patches containing onion extract and allantoin have been demonstrated to be a safe and effective approach in patients with scars of various origins and severity (Karagoz et al., 2009; Prager and Gauglitz, 2018). Up to date, no studies have evaluated the effects of the patch formulation in women underwent more than one Cesarean delivery.

Predicting the consequences of wound healing triggered by dermatological injury can be very difficult. Scars generated by surgery, such as C-section, strongly vary from fine-line and asymptomatic to hypertrophic scars and keloids (Gold et al., 2014).

Nowadays, prevention is considered more appropriate than treatment thus identifying higher-risk individuals, such as women who underwent C-section for the second or more times compared with those without a history of C-section, is the indisputably preferable strategy.

Our study investigated the effects of a patch containing onion extract and allantoin on C-section scars. This patch combines the effect of its ingredients with occlusive action on the scar.

Among topical treatments containing natural compounds, the onion extract is largely used as an anti-scarring agent in several clinical settings, including blepharoplasty scar, dermatological and thoracic post-surgical scar, and after removal of tattoos (Ho et al., 2006; Mylonas and Klaus Friese, 2015; Fang et al., 2017; Owji et al., 2018).

Campanati et al. tested the clinical effect of a gel containing the same ingredients of the patch formulation to treat skin lesions of patients with hypertrophic scars or keloids, concluding that a 24-week application of the gel twice daily reduced erythema and neoangiogenesis. As regards to the other analyzed parameters, such as itching, burning, and relief and the general appearance of the scar, the authors revealed an improvement, although the statistical significance was not reached (Campanati et al., 2010). Regarding C-section scars, a prophylactic use of a gel containing heparin together with onion extract and allantoin was tested at 6 and 12 weeks in women who underwent a Cesarean delivery, for the first time, without including women who had already undergone previous C-section. As in our study, scars assessment was performed by POSAS, and the results showed that the gel was effective to improve the color, stiffness, and irregularity of the scars. However, it is important to underline that these improvements were referred only to the scores of the evaluated patient scale, while no significant changes were found according to the observer Scale (Ocampo-Candiani et al., 2014).

A patch very similar to that evaluated in the present investigation demonstrated beneficial effects for treating post-dermatologic surgery scars. The authors used POSAS for scar assessment and confirmed that an overnight treatment with this patch is safe, well tolerated and able to improve scars’ healing (Prager and Gauglitz, 2018).

These results agreed with those of previous studies in which the gel formulation was successfully used for instance to treat thoracic surgery and for prevention of postoperative peritoneal adhesion (Willital and Heine, 1994; Willital and Simon, 2013). To the best of our knowledge, the present investigation is the first that analyzed the effect of a patch formulation containing Allium cepa and allantoin on subjects who had already undergone previous C-section in respect to women who underwent a Cesarean delivery for the first time. The results allowed us to shed light on the benefits derivable from an intense (overnight) but short-term (just 4 weeks) use of the patch. Notably, by comparison of two experimental groups, considering the number of C-sections, it has been possible to underline a statistically significant improvement in pigmentation, relief and pliability only in women who had already undergone previous C-section.

In comparison with surgical treatments, the topical anti-scarring agents represent a non-invasive and valid alternative. The hypertrophic scars may likely evolve and many subjects do not choose or, more importantly, cannot choose the option of surgical treatment. Also, surgery is often associated to post-surgical complications with consequent polytherapy and adverse events, especially in complicated patients (Poole, 2013; Corbi et al., 2015).

For this reason, it is important to personalize scars management and stimulate the use of preventive and non-invasive methods, especially in subjects at higher risk of developing keloids, ameliorating patients’ compliance and quality of life (Gold et al., 2014).

A significant improvement in pigmentation, relief and pliability was found in the group of women who had already undergone C-section. Regarding the other variables included in the observer scale and all the variables in the patient scale, an improving trend was recorded without reaching statistical significance.

Our results are according to previous studies. By using a device similar to ours, Guertler et al. (Guertler et al.) found that at 3 months, the overnight patch application was able to reduce all the POSAS observer’s scale scores, including pliability and thickness. Moreover, Draelos et al., by using a gel with the same components of the patch used in our study, found that the scar softness was improved at 4 weeks, mainly in the subjects treated with the Onion extract gel (Draelos et al., 2012). In a Chuangsuwanich et al. study (Chuangsuwanich and Jongjamfa, 2014)., a combined herbal extract gel containing Allium cepa was able to reduce the POSAS scores compared with the placebo group in pigmentation, thickness, and overall scores at 12 weeks (all p = 0.04). The POSAS scores in vascularity, relief, and pliability in the treated group were significantly less than those in the placebo group at 8 weeks. Maher et al. also found an improvement in pliability using silicone gel sheets on linear scars (Maher et al., 2012). In our study, the use of a device that combines the above-mentioned properties with a mechanic occlusive effect of the patch could explain the improvements in the POSAS scores that we found.

The natural ingredients of the patch justify the obtained results. In particular, Allium cepa extract has a high content of organo-sulphur compounds and flavonoids that, together with other components such as lectins and vitamins B, C, and E, give reason of its numerous biological and medical activities (Rose et al., 2005). The anti-inflammatory and antimicrobial properties of the Allium cepa have been extensively investigated, and its efficacy in contrasting several microbes including parasites, fungi and bacteria has been demonstrated (Saleheen et al., 2004; Ramos et al., 2006). The antimicrobial property is very important when you consider that during the wound healing inflammation phase, neutrophils and macrophages accumulate with the main aim to prevent infections and modulate inflammation, which, in turn, concurs to delay the healing and increase skin scarring (Tansey and Appleton, 1975; Eming et al., 2007).

Allium cepa can also decrease the histamine levels and improve the collagen organization in pathologic scars (Saulis et al., 2002).

In addition, this extract inhibits the production of cytokines and growth factors, such as IL-6 and VEGF, in fibroblast human cell lines. This activity may play an important role in contrasting the abnormal proliferating process resulting in keloid formation (Pikuła et al., 2014).

The high content in flavonoids, particularly quercetin, justifies also the antioxidant, anti-carcinogenic and anti-mutagenic activities of the Allium cepa that are conferred through several mechanisms (Corzo-Martínez et al., 2007; Hashemzaei et al., 2017).

Allantoin, the other component of the patch used in the present study, has moisturizing and antioxidant properties (Hashemzaei et al., 2017).

High allantoin concentration stimulates keratolytic activity by decreasing the number of cellular junctions in the corneous layer and removing the superficial necrotic elements. Through augmenting keratolytic activity, skin is stimulated to increase self-regeneration (Manca et al., 2016; Selamoglu et al., 2017). These properties are likely responsible for the positive effects of the patch used in this study, that we found especially on pliability.

The wound healing profile induced by allantoin was qualitatively and quantitatively analyzed in female Wistar rats topically treated with an emulsion containing allantoin 5% for 14 days. The assessment of wound area revealed that allantoin ameliorated and accelerated the healing process (Araújo et al., 2010). Allantoin, meanwhile, has been shown to promote cell proliferation (removal of necrotic tissue) and epithelialization and thereby hasten the growth of new healthy tissue. Willital and Heine (Ocampo-Candiani et al., 2014) showed that Allium cepa, on collagen synthesis and its loosening effect on collagen structure, may promote physiological scar development. Because of the presence of a greater collagen substrate, all together these properties could also explain the higher efficacy of the patch in the women who have undergone previous C-section than in the women at the first C-section.

### Strengths and Weaknesses

Strength of the present investigation is the inclusion of two groups of women, differentiated by the number of C-section. Up to date, the effects of a patch containing onion extract and allantoin has never been evaluated on C-section scar and the effectiveness of the gel formulation containing the same ingredients was investigated only in women without a history of Cesarean delivery. In our study, the beneficial effects in pigmentation, relief and pliability have been observed only in the group of women who had already undergone previous C-section, while no significant changes from baseline were found in women who have had a Cesarean delivery for the first time. This finding underlines the importance to personalize and optimize the scar management.

Although the effectiveness of the patch in the group of women with a history of C-section is very evident, the study is limited by the small sample size. A major limitation is also represented by the non-randomized study design. Studies including a larger number of individuals and carried out by comparing the patch with different topical treatments using at least two kinds of scar assessment scales are needed to confirm these results. Moreover, studies with longer follow-up and more evaluation time points, besides randomization, are needed to confirm the beneficial effects of the patch.

Besides, randomized studies comparing the patch with gels or ointments containing the same natural products will be useful to highlight the importance of the patch compressive action on hypertrophic scars and keloids.

## Conclusions

An overnight use of a patch containing Allium cepa extract and allantoin for just 4 weeks was able to improve pigmentation, relief and pliability of C-section scars in women who had undergone previous Cesarean delivery, demonstrating that this topical medical device represents an effective and safe therapeutic approach to manage C-section scars.

## Data Availability Statement

The raw data supporting the conclusions of this article will be made available by the authors, without undue reservation.

## Ethics Statement

The studies involving human participants were reviewed and approved by Comitato Etico Campania Sud (approved number 27_r.p.s.o). The patients/participants provided their written informed consent to participate in this study.

## Author Contributions

All authors read and met Frontiers in Pharmacology criteria for authorship. VC, TI, AF, and MG conceived and designed the study. BC, SL, and LG collected the data. GC and VC performed the statistical analysis and interpretation of the data. VC, GC, and TI wrote the paper. AF and MG critically reviewed the manuscript.

## Funding

This work was funded, in part, by a grant to the University of Salerno from Laboratorio Farmacologico Milanese, LFM (300397CPR18LFM). The authors were not directly compensated for completing this research. Beyond providing support for the project, Laboratorio Farmacologico Milanese, LFM did not contribute to the study design, data collection, data analysis/interpretation, writing of the report, or the decision to submit the article for publication. The industry only provided the funds for a possible open access publication.

## Conflict of Interest

The authors declare that the research was conducted in the absence of any commercial or financial relationships that could be construed as a potential conflict of interest.
