# Supplementary figures and images for: Effectiveness and Tolerability of a Patch Containing Onion Extract and Allantoin for Cesarean Section Scars
Source: Front Pharmacol. 2020 Sep 25;11:569514. doi: 10.3389/fphar.2020.569514 (PMC7546780; doi:10.3389/fphar.2020.569514)

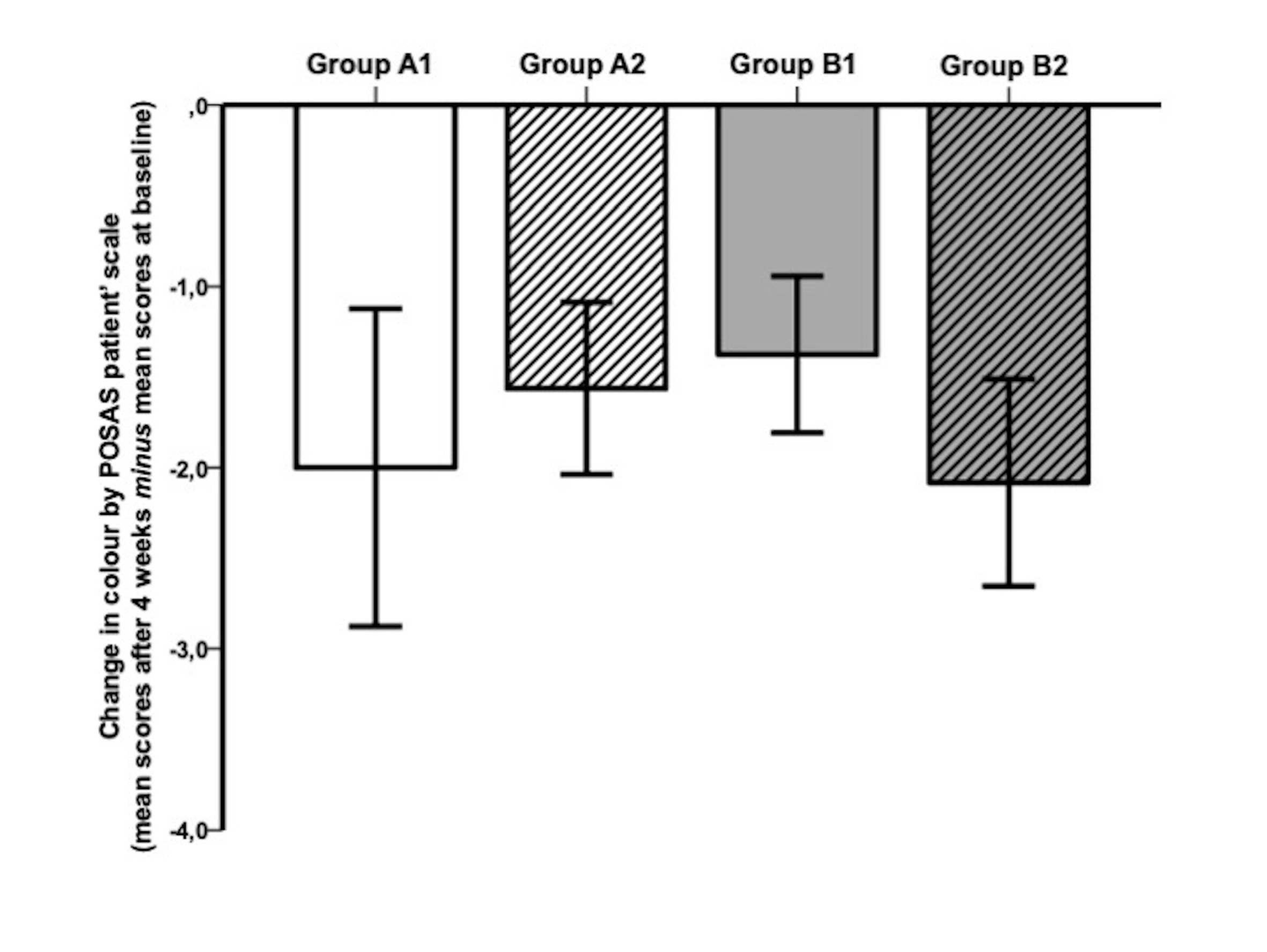

Supplement: Supplementary file 2 [file Image_1.jpeg]

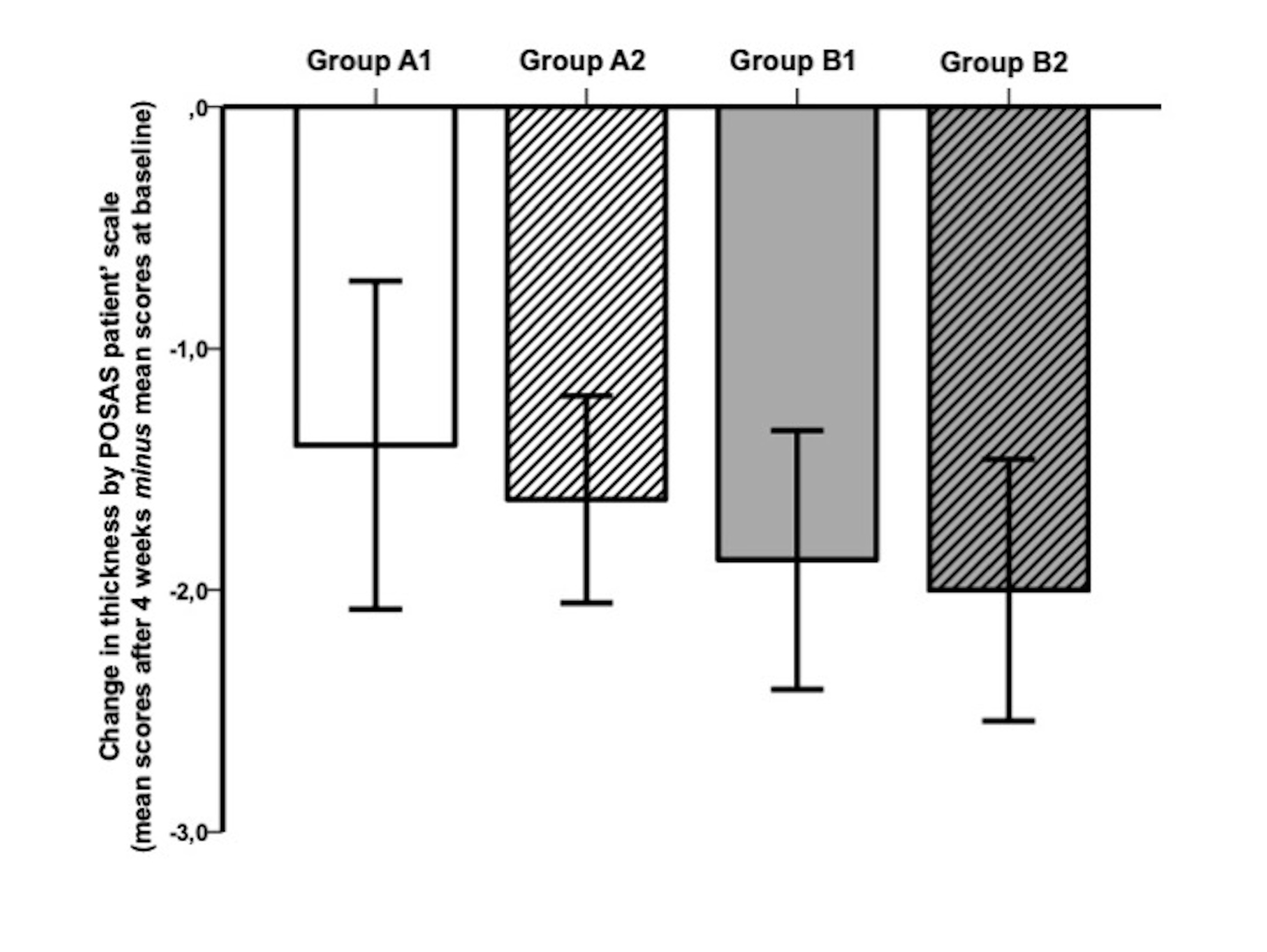

Supplement: Supplementary file 3 [file Image_2.jpeg]

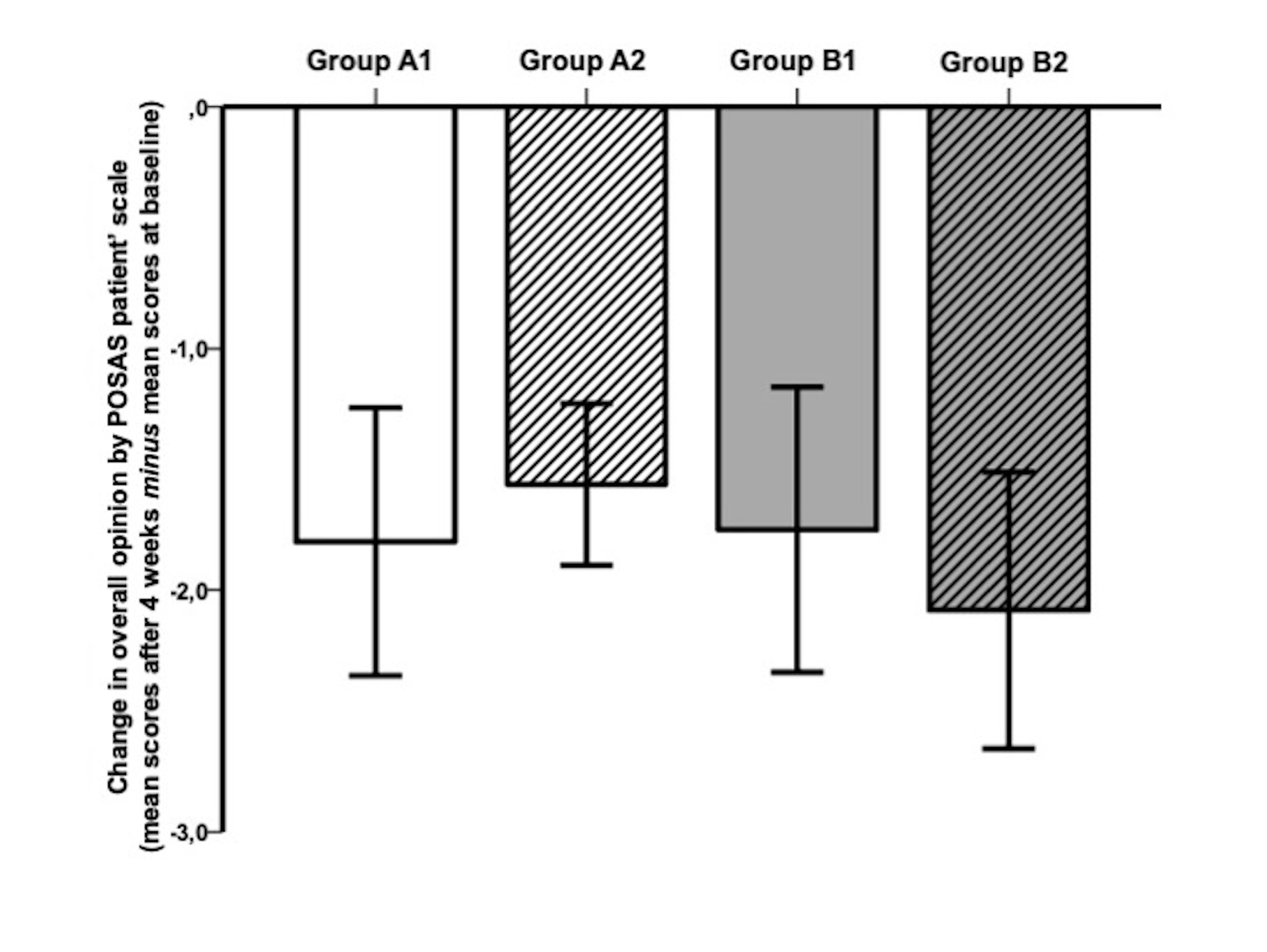

Supplement: Supplementary file 4 [file Image_3.jpeg]

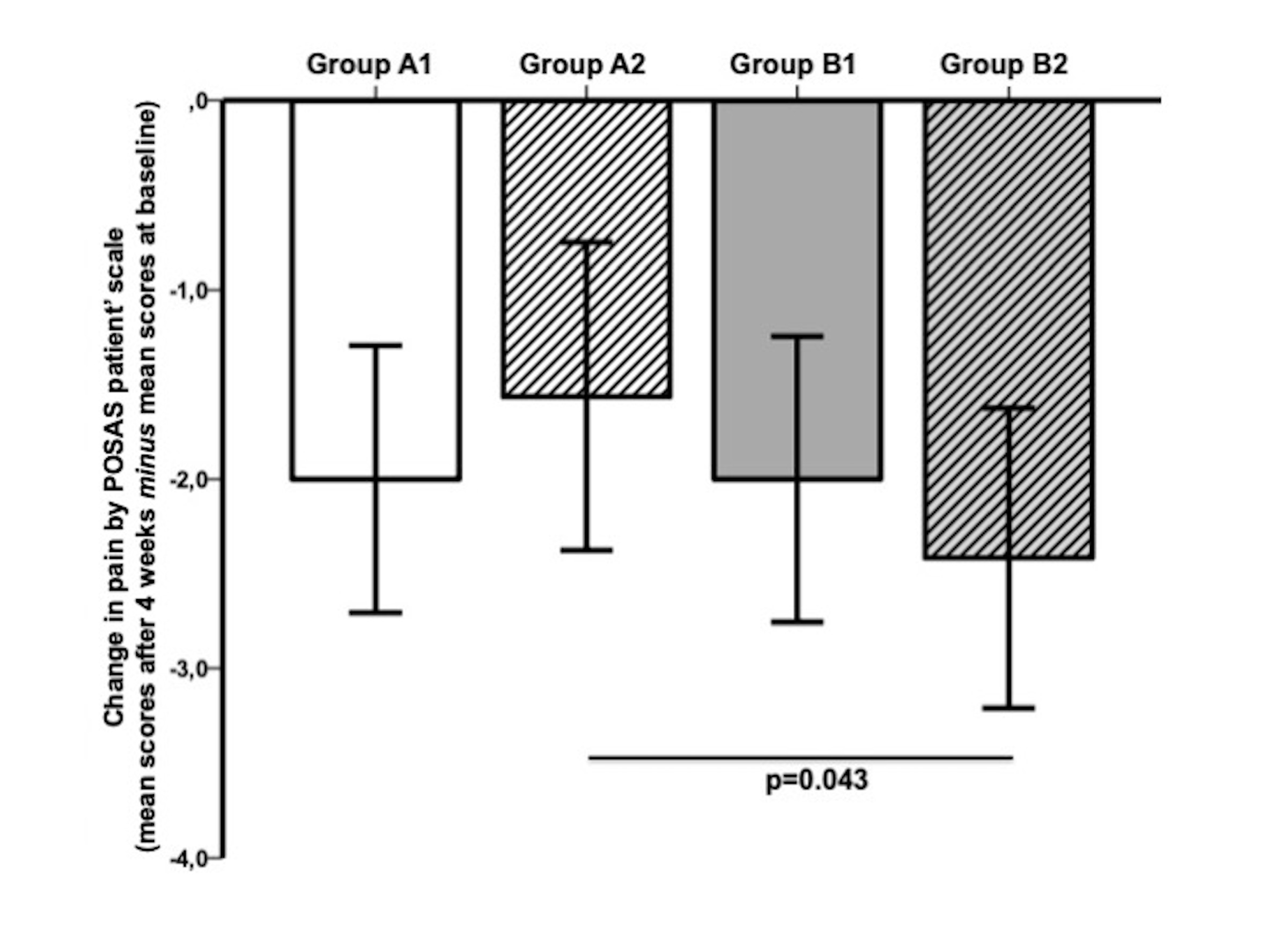

Supplement: Supplementary file 5 [file Image_4.jpeg]

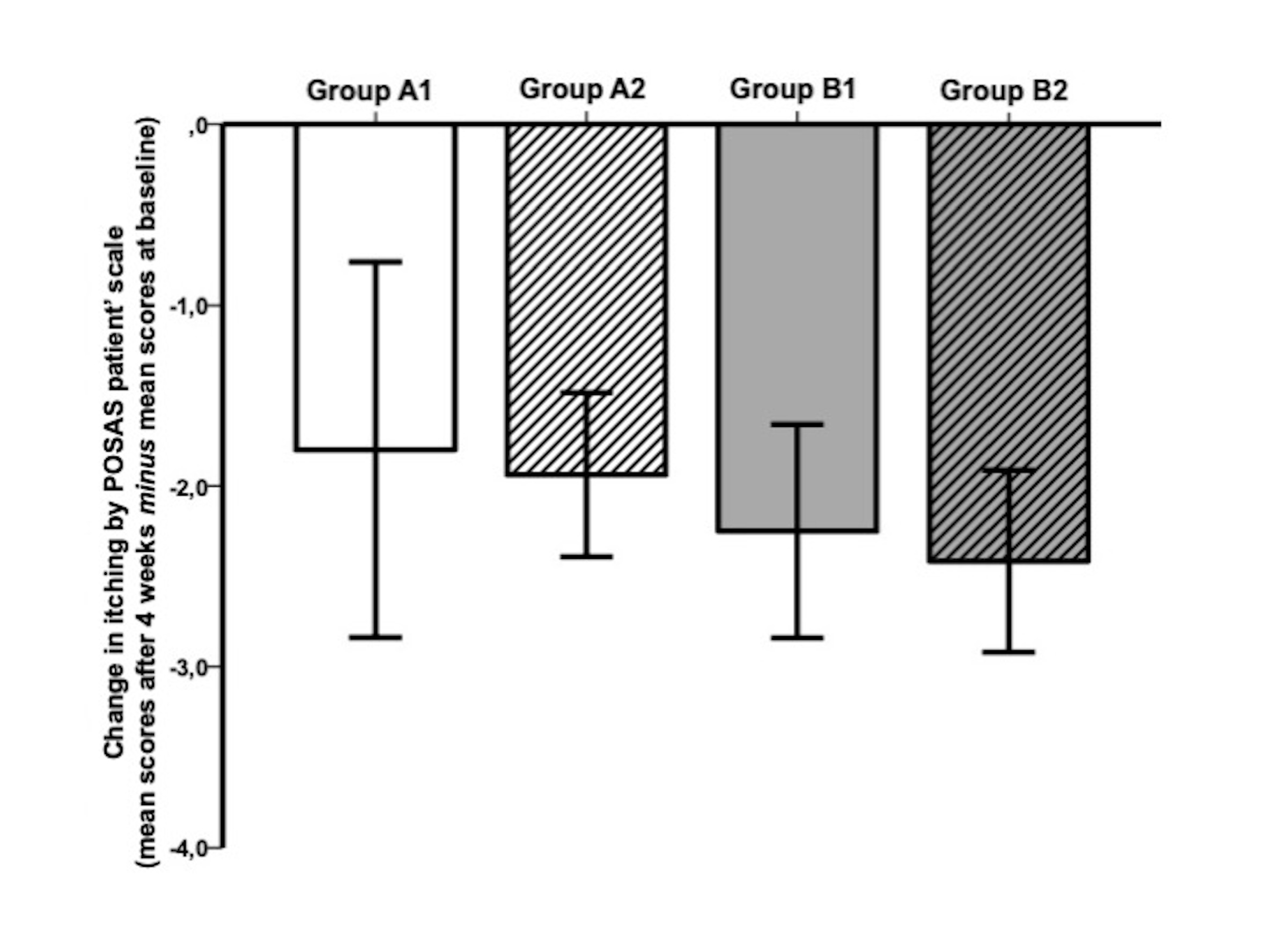

Supplement: Supplementary file 6 [file Image_5.jpeg]

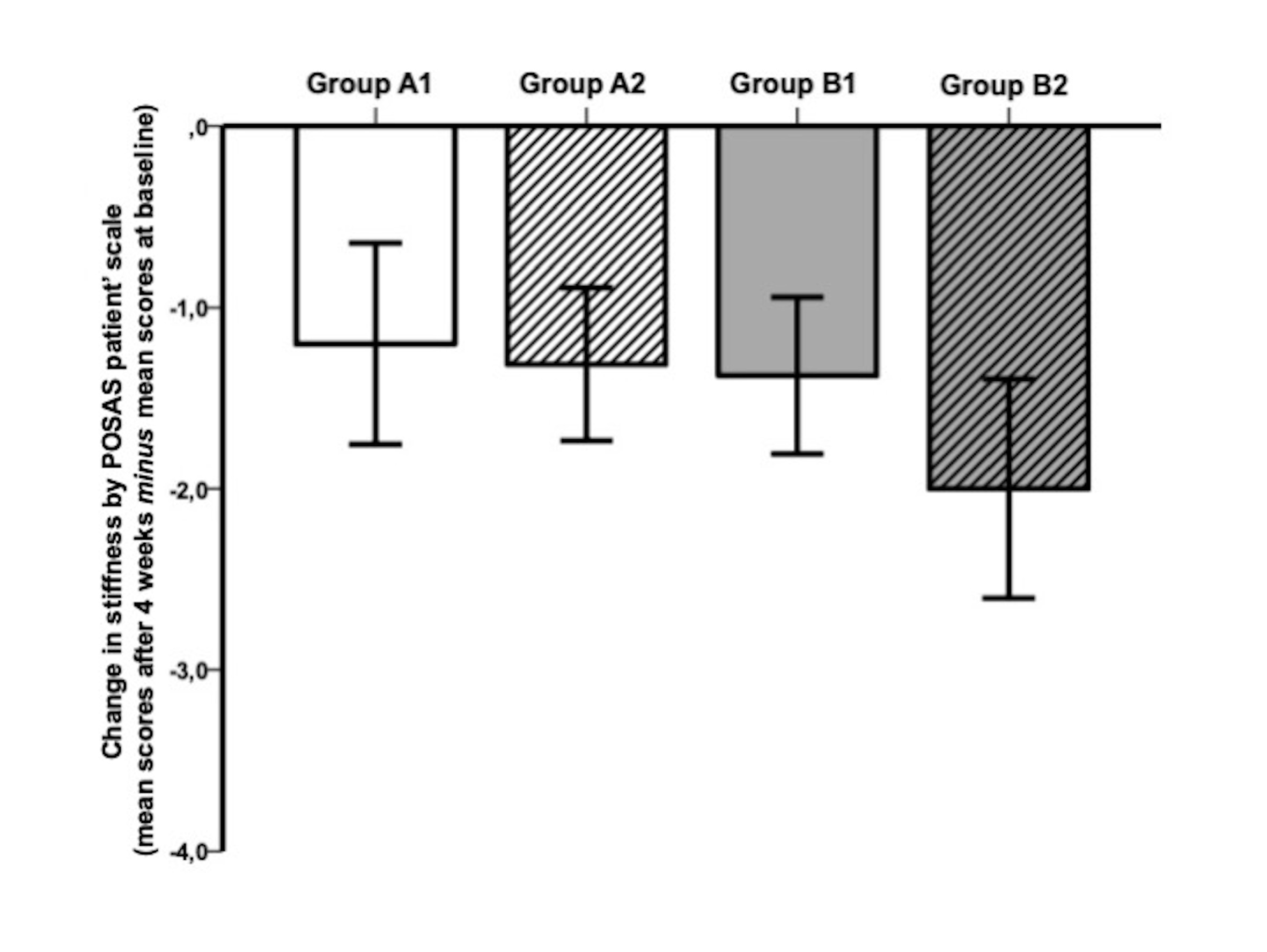

Supplement: Supplementary file 7 [file Image_6.jpeg]

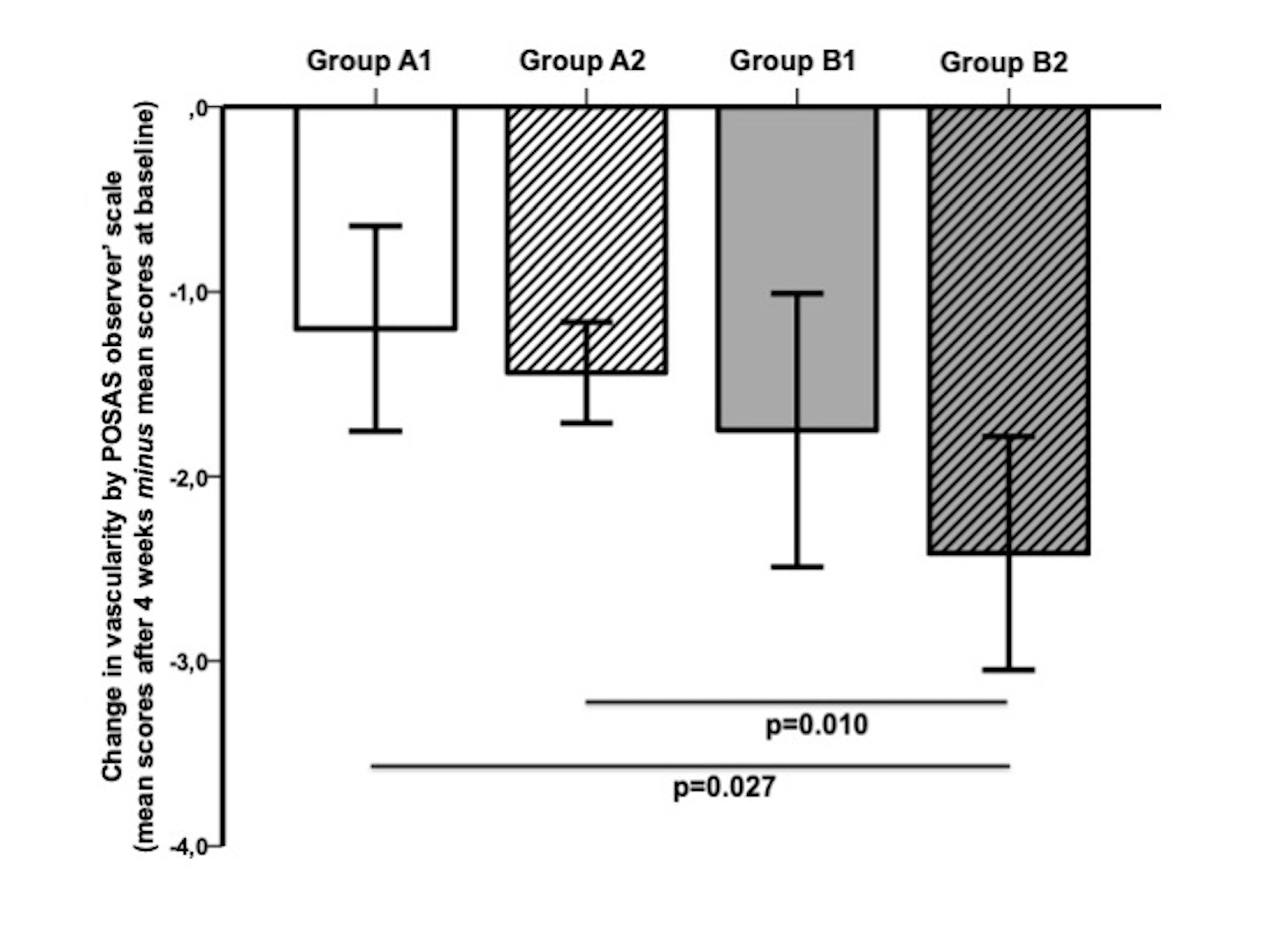

Supplement: Supplementary file 8 [file Image_7.jpeg]

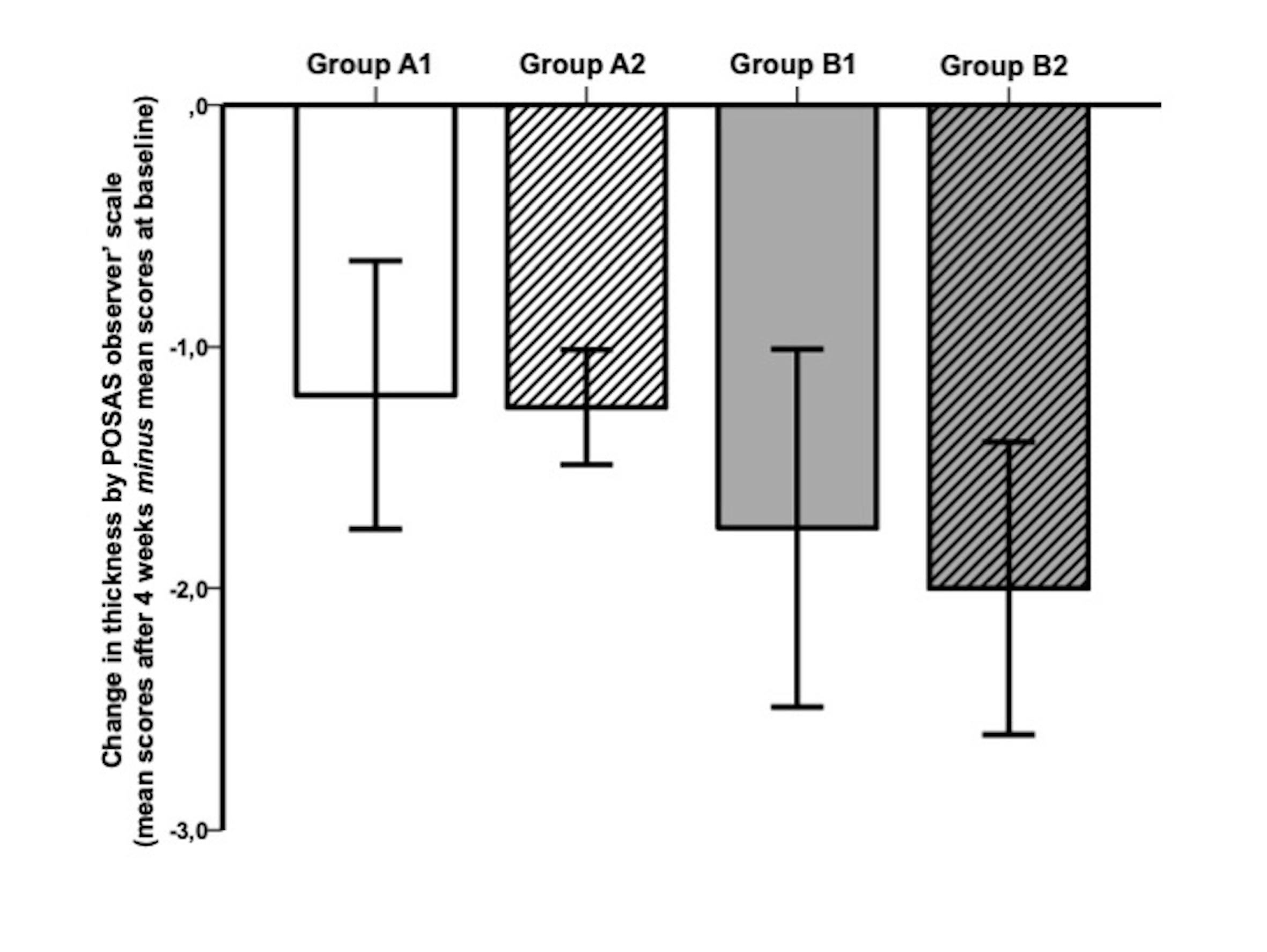

Supplement: Supplementary file 9 [file Image_8.jpeg]

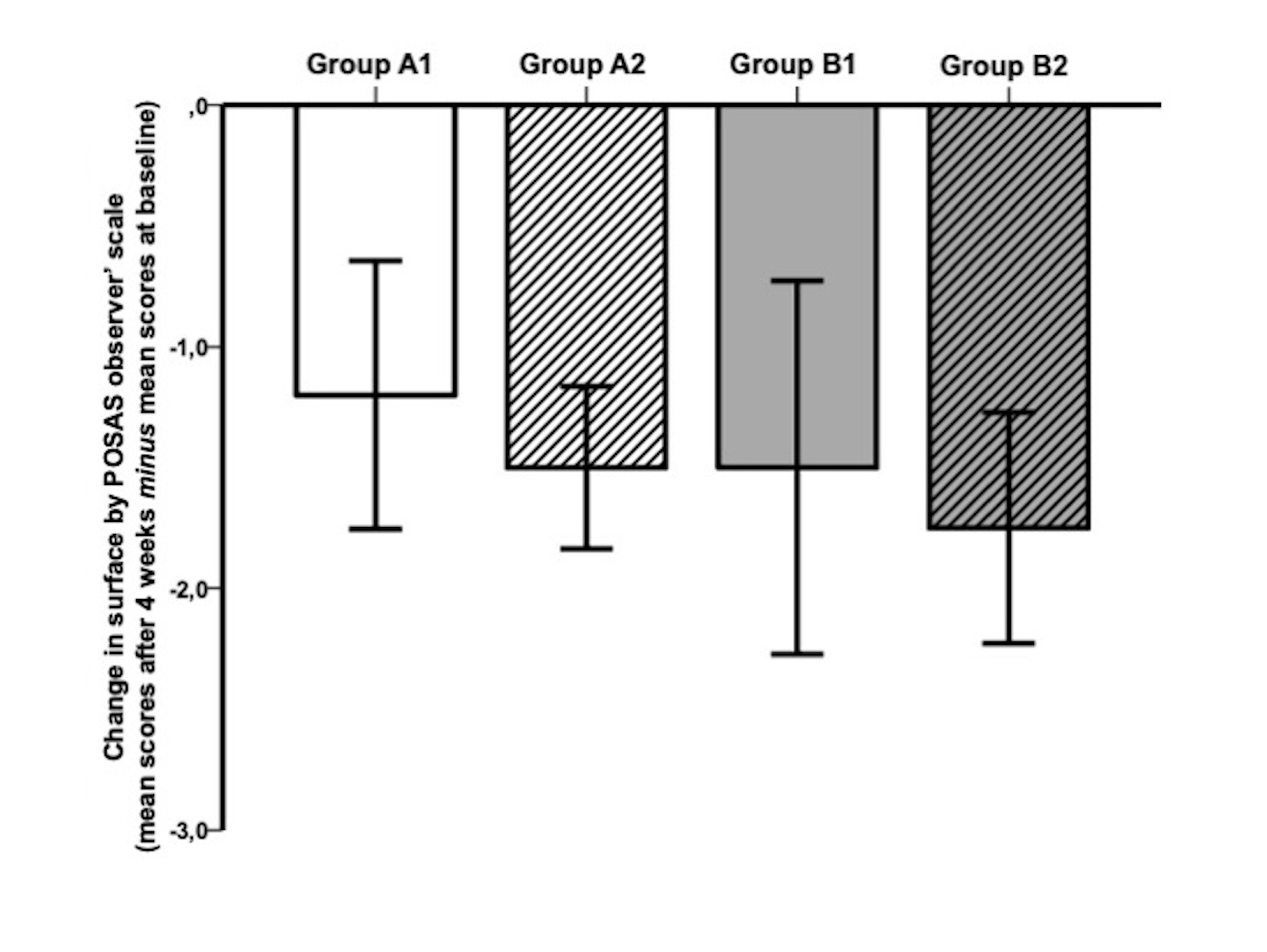

Supplement: Supplementary file 10 [file Image_9.jpeg]
